# Macitentan in the Treatment of Digital Ulcers in Patients with Systemic Rheumatic Autoimmune Diseases: A National Multicenter Study of 42 Patients

**DOI:** 10.3390/jcm14217546

**Published:** 2025-10-24

**Authors:** Miriam Retuerto-Guerrero, Clara Moriano Morales, Ivan Castellvi Barranco, María Hildegarda Godoy Tundido, Clara Méndez Perles, Carlos de la Puente Bujidos, Ana Salome Pareja Martínez, Marta Garijo Bufort, Leyre Riancho Zarrabeitia, Elena Aurrecoechea Aguinaga, Guillermo González Arribas, Esther F. Vicente-Rabaneda, Silvia Montes García, Belén Atienza-Mateo, Vanesa Calvo-Río, Cristina Corrales Selaya, José Andrés Lorenzo Martín, Elvira Díez Álvarez

**Affiliations:** 1 Department of Rheumatology, Complejo Asistencial Universitario de Leon, 24008 Leon, Spain; moriano.c@gmail.com (C.M.M.); elviraleon009@gmail.com (E.D.Á.); 2 Department of Rheumatology, Hospital Universitario de la Santa Creu i Sant Pau, Autoimmune Diseases Functional Unit, 08025 Barcelona, Spain; icastellvi@santpau.cat; 3 Department of Medicine, Universidad Autónoma de Barcelona, 08193 Barcelona, Spain; 4 Department of Rheumatology, Hospital Universitario Puerta de Hierro, 28222 Madrid, Spain; hildagodoy2001@yahoo.es; 5 Department of Rheumatology, Hospital Universitario de Toledo, 45071 Toledo, Spain; clara_mp@hotmail.com; 6 Department of Rheumatology, Hospital Universitario Ramon y Cajal, 28034 Madrid, Spain; delape8@gmail.com; 7 Department of Rheumatology, Hospital Universitario Infanta Leonor, 28031 Madrid, Spain; ana.pareja.martinez@gmail.com; 8 Department of Rheumatology, Hospital de Sagunto, 46520 Valencia, Spain; mgarijobufort@gmail.com; 9 Department of Rheumatology, Hospital de Sierrallana, 39300 Torrelavega, Spain; leyre1987@hotmail.com (L.R.Z.); elena.aurrecoechea@scsalud.es (E.A.A.); 10 Grupo Inmunopatología, Instituto de Investigación Valdecilla (IDIVAL), 39008 Santander, Spain; mateoatienzabelen@gmail.com (B.A.-M.); fiorelfa@hotmail.com (V.C.-R.); c.corrales.selaya@gmail.com (C.C.S.); 11 Department of Rheumatology, Hospital Universitario de A Coruña, 15006 A Coruña, Spain; guillermoarribas93@gmail.com; 12 Department of Rheumatology, Hospital Universitario de la Princesa, 28006 Madrid, Spain; efvicenter@gmail.com; 13 Department of Medicine, Universidad Autónoma de Madrid, 28006 Madrid, Spain; 14 Department of Rheumatology, Hospital Universitario de Talavera de la Reina, 45600 Toledo, Spain; silvia.montes_@hotmail.com; 15 Department of Rheumatology, Hospital Universitario Marqués de Valdecilla, 39008 Santander, Spain; 16 Department of Rheumatology, Hospital Universitario de Burgos, 09006 Burgos, Spain; andrew_1_andres@outlook.com

**Keywords:** Raynaud’s phenomenon, digital ulcers, systemic autoimmune rheumatic diseases, systemic sclerosis, macitentan, endothelin receptor antagonist, efficacy, safety

## Abstract

**Objective:** To evaluate the real-world safety and efficacy of macitentan (MACI) in patients with systemic autoimmune rheumatic diseases (SARDs) and refractory digital ulcers (DUs). **Methods:** We conducted a retrospective observational study of 42 patients treated with MACI (10 mg/day) on a compassionate-use basis across Spanish reference hospitals. Given the cohort’s heterogeneity, a two-step analysis was performed: a global assessment of all patients, followed by a subgroup analysis restricted to those with systemic sclerosis (SSc) or fulfilling very early SSc (VEDOSS) criteria to explore predictors of response. Efficacy was defined as complete healing, partial response, or a lack of response based on physician assessment. Safety was evaluated through analysis of adverse events. **Results:** In the global cohort, MACI demonstrated a high rate of complete ulcer healing (82.9%) at the 3-month follow-up, with a significant reduction in median ulcer count (*p* < 0.001). Subgroup analysis within the SSc/VEDOSS cohort (n = 36) revealed that the presence of gastrointestinal involvement (GI) and a higher baseline DUs were significant predictors of a poorer therapeutic response (*p* = 0.022 and *p* = 0.028). The drug was well-tolerated; adverse events were infrequent and rarely led to treatment discontinuation. **Conclusions:** In this real-world refractory population, MACI was associated with rapid DU healing and a favorable safety profile. GI and higher ulcer burden predicted diminished treatment response in SSc patients. These results support the use of MACI as a valuable therapeutic option for severe digital vasculopathy in SARDs, although further prospective studies are warranted to confirm these observations.

## 1. Introduction

Systemic sclerosis (SSc)-related vasculopathy plays a pivotal role in the initiation and progression of systemic sclerosis, as well as in the development of digital ulcers (DUs) [1]. DUs, which typically occur on the fingers and toes, represent a common and debilitating complication in SSc, often resulting in chronic pain, recurrent infections, and functional impairment [2]. The pathophysiology underlying DUs involves vascular dysfunction, ischemia, and chronic inflammation [3]. Moreover, DUs have been associated with a younger age at disease onset, gastrointestinal involvement, and elevated creatine kinase levels, among other factors [4].

Endothelin-1 (ET-1) serves as a key mediator of vascular hypertrophy, cellular proliferation, inflammation, and fibrosis, and is overexpressed in the plasma of patients with systemic sclerosis, particularly those with DUs and/or pulmonary arterial hypertension (PAH) [5,6]. ET-1 functions as a potent vasoconstrictor and a mild mitogen for arterial smooth muscle cells, acting through ET-1 receptor type A (ETAR) and type B (ETBR). The activation of ETAR promotes vasoconstriction, proliferation, and fibrosis, whereas ETBR mediates vasodilation through the release of nitric oxide and prostaglandins [7]. In SSc, elevated ET-1 levels and overexpression in fibroblasts and endothelial cells correlate with the severity of fibrosis [5]. Furthermore, increased levels of anti-ETAR antibodies have been reported in SSc and are considered predictive and prognostic biomarkers for SSc-associated PAH [8].

Macitentan (MACI), a dual ET-1 receptor antagonist (ERA), is a drug approved for the treatment of PAH, a severe condition characterized by elevated blood pressure within the pulmonary arteries [9]. MACI belongs to a new generation of ERAs distinguished by optimized physicochemical properties that enhance tissue penetration and ensure sustained receptor occupancy compared with earlier agents such as ambrisentan and bosentan (BOSE) [10]. Both MACI and its pharmacologically active metabolite, ACT-132577, possess a long half-life, supporting once-daily dosing and improving patient adherence [11]. The pharmacokinetic profile of MACI is favorable, showing no clinically relevant differences in patients with PAH or in those with renal or hepatic impairment [12]. Furthermore, MACI demonstrates a low potential for clinically significant drug–drug interactions, as it is not a substrate for hepatic transporters, and no elevations in bile salts have been observed in either animal or human studies [13].

The clinical efficacy and safety of MACI were established in the SERAPHIN trial, the first large-scale morbidity and mortality study with long-term outcomes in patients with PAH. This landmark trial demonstrated that MACI significantly reduced morbidity and mortality in both treatment-naïve patients and those receiving background therapy [9]. Based on these findings, MACI has been approved by major regulatory authorities, including the U.S. Food and Drug Administration (FDA), the European Medicines Agency (EMA), SwissMedic, and Health Canada [14].

Based on this pathophysiological framework, MACI represents a promising therapeutic option for addressing microvascular complications such as DUs. The DUAL-1 and DUAL-2 trials were the primary studies assessing MACI for the prevention of SSc-related DUs [15]. These multicenter, randomized, placebo-controlled trials included patients with active or previous DUs and aimed to determine whether MACI could reduce the occurrence of new ulcers. Although both studies failed to meet their primary endpoints, they faced significant criticism for methodological limitations and patient selection biases. Y. Asano [16] emphasized differences in placebo groups and potential social factors influencing the outcomes, suggesting that the DUAL findings should be interpreted with caution and that MACI should remain a therapeutic consideration in SSc.

Beyond its established role in PAH, MACI has attracted growing attention for its potential benefits in treating DUs associated with systemic autoimmune rheumatic diseases (SARDs), particularly SSc. Emerging real-world evidence [17,18,19] suggests that MACI may offer clinical benefits in patients with refractory DUs, including reductions in ulcer recurrence, improved healing, and pain relief, with minimal adverse effects. The present study aims to contribute additional evidence regarding the clinical utility and safety of MACI in refractory cases where conventional treatments have failed.

## 2. Patients and Methods

We conducted a retrospective observational study involving patients under follow-up in rheumatology departments at reference hospitals across Spain. The study aimed to evaluate the safety and efficacy of MACI (10 mg/day) for the treatment of DUs and severe Raynaud’s phenomenon in patients with SARDs. A total of 42 patients treated with MACI on a compassionate-use basis were included. Diagnoses were established using internationally accepted criteria, including the 2013 ACR/EULAR criteria for SSc [20], the 2021 EUSTAR criteria for very early diagnosis of systemic sclerosis (VEDOSS) [21], and other recognized classification criteria for connective tissue diseases.

Organ involvement was confirmed using standardized diagnostic procedures: pulmonary involvement by high-resolution computed tomography (HRCT); gastrointestinal involvement based on clinical manifestations and manometric evaluation; and PAH through right heart catheterization or echocardiography. Antinuclear antibodies (ANA) were detected by indirect immunofluorescence (IIF) on HEp-2 cells and confirmed by immunoblot when appropriate.

Efficacy was defined as complete healing of all existing DUs and the absence of new ulcer formation, partial response as a reduction in the number and/or size of ulcers, and lack of response as no observable improvement. Ulcer healing was assessed by the treating physicians during clinical evaluation and not by patient self-reporting clinical evaluation, rather than patient self-report, as per previously published definitions. Safety was evaluated through systematic documentation and analysis of adverse events (AEs).

### 2.1. Statistical Analysis

Descriptive statistics were used to summarize patient demographics and baseline characteristics. The distribution of continuous variables was assessed using the Shapiro–Wilk test. Variables with a normal distribution are presented as mean (standard deviation, SD) and were compared using Student’s *t*-test for paired samples. Non-normally distributed variables are expressed as median with interquartile range (IQR) and were compared using the Mann–Whitney U test. Categorical variables were presented as percentages and compared using Chi-square tests, with Fisher’s exact test applied when expected values were less than five. The primary outcome of this study was the improvement in DU healing, categorized into total resolution, partial resolution, or ineffectiveness. A *p*-value of less than 0.05 was considered statistically significant. All analyses were performed using SPSS version 25 (IBM Corp., Armonk, NY, USA).

### 2.2. Ethical Concerns

This study was conducted in adherence to ethical guidelines and was approved by the Ethics Committee of Complejo Asistencial Universitario de Leon (protocol code 24304, 26 November 2024). Patient privacy and confidentiality were maintained throughout the study in accordance with the General Data Protection Regulation (GDPR) and other applicable regulations. The ethical principles outlined in the Declaration of Helsinki were strictly followed, ensuring that patient welfare was prioritized at every stage of the research process. Furthermore, all adverse events were carefully documented, and appropriate measures were taken to mitigate any potential harm to patients.

## 3. Results

42 patients were recruited, with a majority being female (78.6%) and a mean age of 64.1 (SD 11.4) years at the initiation of MACI therapy. Three patients had not developed DUs but experienced severe, painful Raynaud’s phenomenon and/or ischemic lesions that did not progress to ulceration. The most frequent SARD in our cohort was SSc, with 31 patients classified as limited cutaneous SSc and 6 as diffuse cutaneous SSc (61.9% anticentromere antibody positivity). Additionally, 2 patients fulfilled VEDOSS criteria, 1 was diagnosed with undifferentiated connective tissue disease (UCTD), 1 with antiphospholipid syndrome (APS), and 1 with mixed cryoglobulinemia.

Seven patients were active smokers and 11 were former smokers; 12 had exposure to cold either occupationally and/or environmentally. One patient suffered from PAH. With respect to DU burden, 71.4% of patients exhibited fewer than five lesions, 21.4% had between five and ten, and 7.2% presented with more than ten ulcers. The median number of DUs per patient was 2 (IQR 1–5). Regarding anatomical distribution, DUs were limited to the hands in 73.8% of cases, whereas 26.2% involved both the hands and feet. The mean age at the onset of the first DU was 57.5 years (SD 13), and the median duration from the appearance of the first ulcer to the initiation of MACI treatment was 35.8 months (IQR 12.4–128). Demographic, clinical and immunological characteristics are summarized in Table 1.

### 3.1. Previous Therapies and Treatment Regimens

Prior to receiving MACI, the majority of patients had undergone various treatment regimens, including calcium channel blockers (CCBs) in 39 patients (92.9%), phosphodiesterase type 5 inhibitors (PDE5i) in 20 patients (47.6%), intravenous prostacyclins (PC) in 19 patients (45.2%), ambrisentan in 3 patients (7.1%) and BOSE in 40 patients (95.2%). The reasons for discontinuing previous therapies included adverse events (AEs) in 25 out of 40 patients who had been treated with BOSE, and ineffectiveness in 16 out of 19 patients who had received prostanoid therapy. At the time of initiating MACI, 15 patients (35.7%) required antibiotics due to infections associated with their ulcers, 3 patients (7.1%) had undergone axillary sympathectomy, and 7 patients (16.7%) had undergone amputations due to severe, non-healing ulcers.

At the time of MACI initiation, 13 patients were receiving calcium channel blockers (CCBs), 3 were on phosphodiesterase type 5 inhibitors (PDE5i), and 2 were treated with intravenous prostacyclin (PC). Six patients received combination therapy with two vasodilators (4 with CCBs plus PDE5i, and 2 with CCBs plus PC), while one patient was on a triple vasodilator regimen (CCB, PDE5i, and PC). Seventeen patients received MACI as monotherapy. In addition, 22 patients were also taking aspirin as part of their treatment regimen.

### 3.2. Efficacy Evaluation

**Figure 1:**
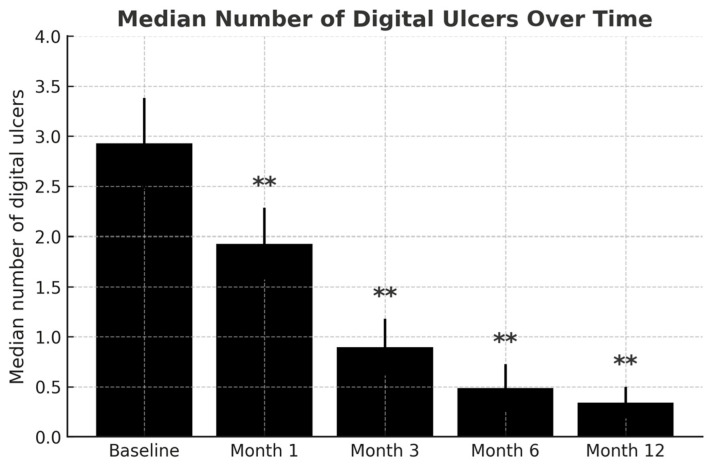
Median number of digital ulcers at baseline and follow-up time points (1, 3, 6, and 12 months). ** *p* < 0.001 compared to baseline.

At the 3-month follow-up, efficacy was evaluated in 41 patients (one patient was excluded due to early adverse events). The results showed a complete healing of DUs in 34 patients (82.9%), partial improvement in 4 patients (9.8%), and no improvement in 3 patients (7.3%). The median number of ulcers decreased progressively throughout the study: from 1 (IQR 0–3) at one month to 0 (IQR 0–1.25) at three months, and 0 (IQR 0–0) at six months (*p* < 0.001) (Figure 1). To further explore the role of concomitant therapies, patients were stratified into monotherapy versus combination therapy (dual, triple, or quadruple regimens). Efficacy, defined as complete healing compared to partial or no response, did not differ significantly between the groups (*p* = 0.93). Notably, none of the three patients who presented with pre-ulcerative ischemic lesions progressed to digital ulceration during follow-up.

**Figure 2:**
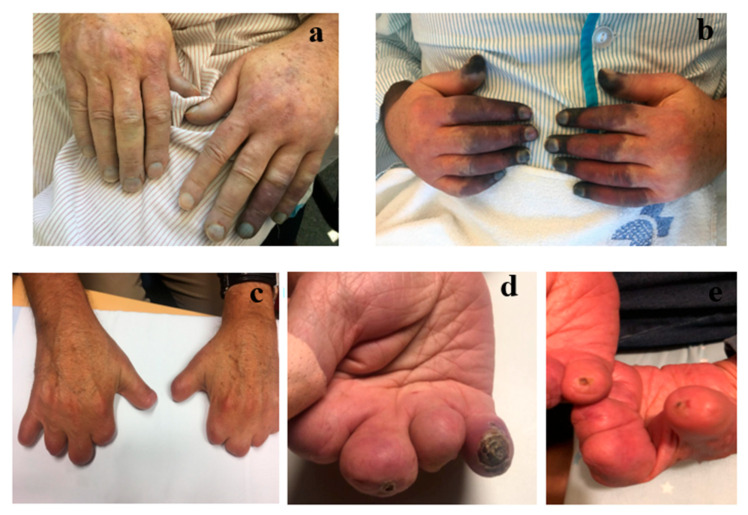
Evolution of digital ulcers in a patient diagnosed with cryoglobulinemia. (**a**) Day 3 of hospitalization in the Vascular Surgery department due to severe Raynaud’s phenomenon and fixed cyanosis. (**b**) Day 7, critical ischemia refractory to prostanoids; MACI 10 mg/day is initiated, and amputation of the middle and distal phalanges is performed. (**c**) After 3 years of treatment with MACI, no new ulcers develop, leading to treatment discontinuation. (**d**) Ulcer recurrence on the stump, prompting the reintroduction of MACI 10 mg/day. (**e**) One and a half months later, follow-up shows near-complete ulcer healing.

In Figure 2 the clinical course of DUs in a patient diagnosed with cryoglobulinemia is illustrated, demonstrating the progression from severe ischemic manifestations and surgical intervention to therapeutic response with MACI, subsequent remission, relapse, and reinitiation of treatment leading to ulcer healing. Of the 5 patients receiving PC at the start of MACI, it was possible to discontinue treatment in 3, while the other 2 patients only required occasional doses; PC was not initiated in new cases. One patient discontinued MACI because of total lack of efficacy during the observation period, which had a mean duration of 36.8 (SD 28.1) months.

A subanalysis was performed in the majority subgroup of patients with SSc and those fulfilling VEDOSS criteria, who had received MACI for at least 3 months, in order to identify potential clinical associations with treatment response (n = 36). Patients with gastrointestinal involvement (100% vs. 46.6%, *p* = 0.022) and those with a higher number of ulcers at the start of treatment [6.3 (SD 4.6) vs. 2.2 (SD 2), *p* = 0.028] showed a less favorable response to MACI (total response versus partial response/ineffectiveness). No differences were observed in the response to MACI in relation to other clinical manifestations, analytical characteristics or the time elapsed since the appearance of the first DUs (Table 2).

**Figure 3:**
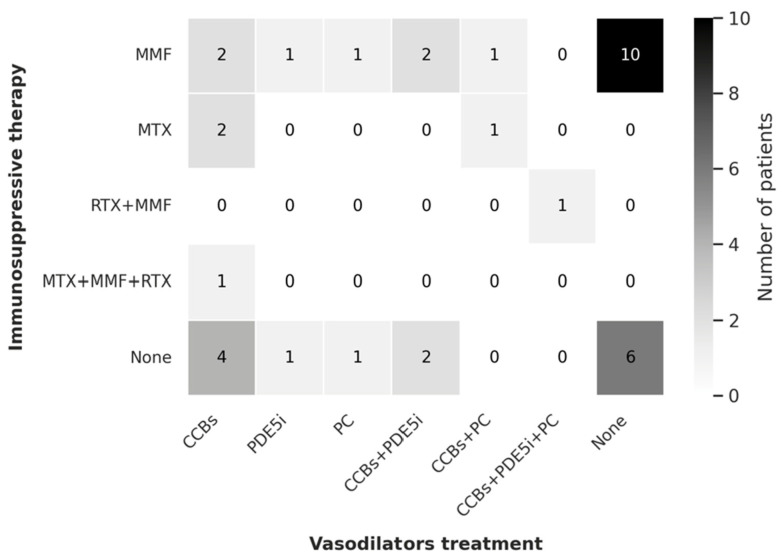
Heatmap of concomitant treatments with MACI. CCBs, calcium channel blockers; MMF, mycophenolate mofetil; MTX, methotrexate; PC, intravenous prostacyclins; PDE5i, phosphodiesterase type 5 inhibitors; RTX, rituximab.

Concomitant immunosuppressive therapy was administered to 22 patients (61.1%). Mycophenolate mofetil was the most frequently used agent (n = 17), followed by methotrexate (n = 3). One patient received a combination of mycophenolate mofetil and rituximab, and another was treated with triple immunosuppressive therapy including rituximab, mycophenolate mofetil, and methotrexate. Additionally, low-dose prednisone (≤5 mg/day) was prescribed in 12 patients. The distribution of concomitant immunosuppressive and vasodilator regimens in patients treated with MACI is summarized in Figure 3.

Upon stratifying patients by disease severity, defined by prior need for antibiotic therapy, surgical sympathectomy, or digital amputation, MACI demonstrated a consistent therapeutic effect across all subgroups. Patients with severe disease responded similarly to those with milder forms, as evidenced by non-significant differences in outcomes among those with prior sympathectomy and/or amputation (*p* = 0.177), or antibiotic use (*p* = 0.157). In contrast, among patients who had previously received BOSE, the drug was associated with significantly reduced efficacy in these high-risk subpopulations, with marked declines in clinical response (*p* = 0.047, and *p* < 0.001, respectively). The efficacy of MACI was evaluated in the patients who had previously received BOSE. No significant differences in MACI response were observed (*p* = 0.181), with efficacy demonstrated in 78.6% (11/14) of patients who discontinued BOSE due to ineffectiveness and 86.4% of those who discontinued it because of adverse events (19/22).

### 3.3. Safety Profile

In terms of safety, MACI was generally well tolerated by the majority of patients. A total of four patients (9.5%) experienced adverse events (AEs) that required permanent discontinuation of treatment, including peripheral edema in the lower extremities (n = 2), palpitations (n = 1), and pruritus (n = 1). One additional patient temporarily discontinued therapy during the summer months due to lower-limb edema. Notably, no patient developed liver enzyme elevation or anemia secondary to MACI treatment. No serious AEs directly related to MACI were observed. One patient unfortunately died from lung adenocarcinoma during the follow-up period, which was deemed unrelated to treatment. Importantly, only one patient developed PAH, and none of the patients fulfilling VEDOSS criteria progressed to definite SSc during the study period.

## 4. Discussion

DUs are a common and debilitating complication in patients with SARDs. Although the DUAL-1 and DUAL-2 trials [15] did not demonstrate significant efficacy for MACI in preventing new DUs, its therapeutic role has been suggested in refractory cases through several clinical reports [19,20,21]. Our study provides crucial real-world evidence in this highly refractory population, which is often excluded from randomized controlled trials (RCTs). In contrast to the DUAL trials, our cohort consisted of patients treated under compassionate use with a focus on healing active ulcers rather than prevention. Furthermore, conducting a conventional RCT in this specific population—comprising patients who have failed multiple lines of therapy, including BOSE and intravenous prostacyclins—poses significant ethical and practical challenges. Our findings thus help fill an important evidence gap by demonstrating a favorable clinical response, including rapid healing of active ulcers within three months, a reduction in the need for rescue prostacyclin therapy, and consistent efficacy even in patients who had previously failed multiple treatments. These results, aligning with previous case series, support MACI as a valuable treatment option in complex, real-world settings and contribute to the growing body of evidence for its use in managing refractory DUs.

We observed a favorable clinical response even in patients who had previously failed multiple lines of therapy. Moreover, MACI was associated with rapid (within less than 3 months) healing of active DUs, with no recurrence observed. Importantly, its use also correlated with a reduction in both the number of patients requiring prostacyclin therapy and the number of prostacyclin infusion cycles. These results are consistent with those reported in previously published case reports and case series [17,18,19] further supporting MACI as a valuable treatment option in refractory cases.

In our SSc subgroup analysis, treatment response to MACI varied based on clinical features, most notably with poorer outcomes observed in patients with gastrointestinal involvement. These results align with previous reports linking gastrointestinal manifestations to an increased risk of refractory DUs and disease chronicity [4]. Furthermore, in our cohort, a higher baseline ulcer burden at the initiation of MACI therapy was associated with poorer therapeutic response and higher recurrence rates. These findings are consistent with the DUO Registry, which identified a greater initial ulcer load as a predictor of both increased frequency and prolonged persistence of DUs. This reinforces the prognostic value of baseline ulcer count as an indicator of disease severity and therapeutic resistance, highlighting the importance of early and aggressive intervention in patients with extensive ulcerative involvement [22].

DUs in SSc generate a significant economic burden due to the direct costs of hospitalization, specialized treatments, and associated complications [23]. Additionally, indirect losses from reduced work productivity and disability contribute to the overall financial impact. Hospitalization for DUs treatment, including intravenous prostacyclins administration, represents a remarkable financial burden due to inpatient care, prolonged hospital stays, and associated complications. The implementation of rescue therapies, such as MACI, could reduce these costs by preventing recurrence and improving patient quality of life. Our results suggested that MACI, by preventing ulcer recurrence, reduce the necessity for intravenous prostacyclins, thereby minimizing hospital admissions and associated complications.

No safety-related concerns were observed in our cohort. MACI’s safety is well-established and supported by large-scale trials which confirmed its long-term efficacy and tolerability in PAH [9]. Real-world studies report minimal adverse effects, such as mild peripheral edema and headache, rarely leading to discontinuation, with no significant liver enzyme elevations. Additionally, MACI’s favorable pharmacokinetic profile minimizes drug–drug interactions, unlike BOSE, which induces cytochrome P450 enzymes, reducing PDE5 inhibitor efficacy (e.g., sildenafil, tadalafil) and interacting with immunosuppressants like cyclosporine, increasing toxicity risks [24]. In our cohort, no pharmacological interactions or hepatic alterations were observed, consistent with previous findings and further reinforcing MACI’s favorable safety profile in clinical practice. Additionally, our indirect comparison between both treatments suggests a relative advantage of MACI in patients with greater clinical complexity.

Although the majority of patients in our cohort had SSc, some presented with UCTD, APS, or cryoglobulinemia, introducing heterogeneity. Nevertheless, MACI may provide therapeutic benefits as a concomitant therapy alongside antiplatelets, anticoagulants, and/or immunosuppressants. In APS, where endothelial dysfunction and thrombosis predominate due to antiphospholipid antibodies, MACI could improve distal perfusion and protect the endothelium, complementing anticoagulation without replacing it. In cryoglobulinemia, a condition characterized by immune-complex–mediated vasculitis and endothelial injury, ET-1 receptor antagonism may promote vasodilation, facilitate ulcer healing, and attenuate secondary endothelial activation when used in combination with immunosuppressive therapy. This potential mechanism is consistent with findings from a reported case demonstrating successful treatment with BOSE for DUs associated with mixed cryoglobulinemia [25].

Therefore, despite the heterogeneity of the cohort, which limits generalizability, our findings suggest that MACI may serve as a valuable adjunctive therapy, promoting vascular perfusion and ulcer healing across various SARDs affecting the microvasculature. This study, however, has several limitations. First, its retrospective and observational design, the absence of a control or comparator arm, and the presence of concomitant therapies may have introduced confounding factors, thereby limiting the ability to establish causal relationships between MACI and the observed clinical outcomes. Second, although most patients had SSc, the inclusion of individuals with UCTD, APS, and cryoglobulinemia introduces heterogeneity due to differences in underlying pathophysiology and vascular risk, potentially affecting the interpretation of treatment efficacy. Third, the relatively small sample size and compassionate-use setting may have favored the inclusion of patients with more severe or refractory disease, while the lack of systematic collection of clinically relevant variables—such as the modified Rodnan skin score or a standardized DU severity/area index with blinded photographic adjudication—further limits phenotypic characterization, external comparability, and overall generalizability.

Despite these limitations, the study presents several notable strengths. It represents one of the largest real-world cohorts to date evaluating MACI for refractory DUs, with the prolonged follow-up and systematic documentation of both efficacy and safety outcomes. The consistent therapeutic responses observed across patients with severe disease, including those who had failed multiple prior therapies, underscore the clinical relevance of MACI in this difficult-to-treat population. Importantly, although recent evidence-based guidelines for the management of DUs in SSc do not currently recommend MACI as a therapeutic or preventive option [26], our findings suggest that ET-1 receptor antagonism with MACI may provide a promising adjunctive strategy in carefully selected patients with refractory disease. By demonstrating consistent efficacy and safety in this challenging population, the present study generates hypotheses and provides a strong rationale for future prospective, adequately powered randomized trials to further evaluate the potential role of MACI in SSc-related vascular complications and other SARDs.

## 5. Conclusions

In summary, our results, along with those previously reported, suggest that MACI may represent a promising therapeutic option for treating severe DUs refractory to conventional therapies. We observed favorable clinical responses, even in patients who were non-responders to BOSE and those with a history of critical ischemia. However, these observations should be interpreted with caution given the negative results of DUAL-1 and DUAL-2, and should be considered hypothesis-generating rather than definitive evidence of efficacy.

## Figures and Tables

**Table 1:** Demographic, clinical, immunological characteristics and prior treatments of 42 patients treated with MACI.

	n 42
**Demographics** Female, n (%) Age at MACI onset Age at onset of Raynaud’s, years Age at first DU diagnosis, years Time from first DU diagnosis to MACI, years	33 (78.6) 64.1 (SD 11.4) 51.4 (SD 14.4) 57.5 (SD 13) 3 (1.1–10.9)
**Disease classification** Diffuse SSc, n (%) Limited SSc, n (%) VEDOSS, n (%) Other SARDs, n (%)	6 (14.3) 31 (73.8) 2 (4.8) 3 (7.1)
**Autoantibody Profile** ANA positive, n (%) ACA positive, n (%) Anti-SCL-70 positive, n (%) Antiphospholipid ab positive, n (%)	38 (90.5) 26 (61.9) 7 (16.7) 2 (4.8)
**Smoking behavior** Current, n (%) Former, n (%)	7 (16.7) 11 (26.2)
**DU count at baseline** <5 ≥5–10 >10 Median total DU count	30 (71.4) 9 (21.4) 3 (7.2) 2 (1–5)
**DU localization**—Hands only, n (%)	31 (73.8)
**Capillaroscopic features** Cutolo “early” pattern Cutolo “active” pattern Cutolo “late” pattern Normal or non-specific	11 (26.2) 9 (21.4) 16 (38.1) 6 (14.3)
**PAH, n (%)**	1 (2.4)
**Previous therapy** Calcium antagonist PDE5 inhibitor Ambrisentan Bosentan Intravenous prostanoids Antibiotics Acetylsalicylic acid Amputation Surgical sympathectomy Systemic therapy	39 (92.9) 20 (47.6) 3 (7.1) 40 (95.2) 19 (45.2) 15 (35.7) 22 (52.4) 7 (16.7) 3 (7.1) 23 (54.8)

Data represent median (IQR), mean (SD) or number (%). Abbreviations: ab, antibody; ACA, anticentromere antibody; ANA, antinuclear antibody; Anti-SCL-70, anti-topoisomerase I antibody; DU, digital ulcers; MACI, macitentan; SARDs, systemic autoimmune rheumatic diseases; SSc, systemic sclerosis; VEDOSS, very early diagnosis of systemic sclerosis.

**Table 2:** Clinical associations with response to MACI in SSc and VEDOSS patients treated for at least 3 months.

	Total Response n 30	Partial/No Response n 6	*p* Value
Female, n (%)	25 (83.3)	4 (66.7)	0.573
dcSSc, n (%)	25 (83.3)	3 (50)	0.109
Mean total DU count	2.2 (SD 2)	6.3 (SD 4.6)	**0.028**
ANA, n (%) ACA, n (%)	29 (96.7) 20 (66.6)	6 (100) 3 (50)	1 0.645
Telangiectasias, n (%)	22 (73.3)	6 (100)	0.302
Calcinosis, n (%)	20 (66.6)	3 (50)	0.645
Joint involvement, n (%)	8 (26.7)	3 (50)	0.343
GI, n (%)	14 (46.6)	6 (100)	**0.022**
ILD, n (%)	16 (53.3)	3 (50)	1
Late capillar. pattern, n (%)	11 (36.7)	4 (66.7)	0.367
MACI mono (no vaso.), n (%)	16 (53.3)	5 (83.3)	0.367
CS use, n (%)	8 (26.7)	3 (50)	0.343
IS therapy, n (%)	16 (53.3)	3 (50)	1
Symp. and/or amput., n (%)	3 (10)	2 (33.3)	0.180

Data represent median (IQR), mean (SD), or number (%). Abbreviations: ACA, anticentromere antibody; amput, amputation; ANA, antinuclear antibody; CS, corticosteroids; dcSSc, diffuse cutaneous systemic sclerosis; DU, digital ulcer; GI, gastrointestinal; ILD, interstitial lung disease; IS, immunosuppressants; MACI, macitentan; mono, monotherapy; symp., sympathectomy, vaso., vasodilator.

## Data Availability

The data supporting the findings of this study are available from the corresponding author upon reasonable request. All data generated or analyzed during this study are included in this published article.

## Abbreviations

The following abbreviations are used in this manuscript:

ACA	anticentromere antibody
ACR	American College of Rheumatology
AE	adverse event
APS	antiphospholipid syndrome
CCB	calcium channel blocker
DU	digital ulcer
EMA	European Medicines Agency
ERA	endothelin receptor antagonist
ET-1	endothelin-1
ETAR	endothelin receptor type A
ETBR	endothelin receptor type B
EULAR	European Alliance of Associations for Rheumatology
EUSTAR	EULAR Scleroderma Trials and Research
FDA	U.S. Food and Drug Administration
GDPR	General Data Protection Regulation
IQR	interquartile range
MACI	macitentan
MMF	mycophenolate mofetil
PAH	pulmonary arterial hypertension
PC	intravenous prostacyclins
PDE5i	phosphodiesterase type 5 inhibitors
RCT	randomized controlled trial
RTX	rituximab
SARD	systemic autoimmune rheumatic disease
SD	standard deviation
SSc	systemic sclerosis
UCTD	undifferentiated connective tissue disease
VEDOSS	very early diagnosis of systemic sclerosis
